# Ustekinumab in Ulcerative Colitis: A Real-Life Effectiveness Study Across Multiple Belgian Centers (SULTAN)

**DOI:** 10.3390/jcm14186506

**Published:** 2025-09-16

**Authors:** Tom Holvoet, Marie Truyens, Catherine Reenaers, Filip Baert, Stijn Vanden Branden, Anneline Cremer, Lieven Pouillon, Pieter Dewint, Wouter Van Moerkercke, Jean-François Rahier, Liv Vandermeulen, Jurgen Van Dongen, Harald Peeters, Guy Lambrecht, Anne Vijverman, Amber Van Haecke, Anne Hoorens, Triana Lobaton

**Affiliations:** 1 Department of Gastroenterology, UZ Gent, Corneel Heymanslaan 10, 9000 Gent, Belgium; tom.holvoet@uzgent.be (T.H.); marie.truyens@uzgent.be (M.T.); 2 Department of Gastroenterology, VITAZ, 9100 Sint Niklaas, Belgium; 3 Department of Internal Medicine and Pediatrics, Gent University, 9000 Gent, Belgium; 4 Department of Gastroenterology, CHU Liege, 4000 Liege, Belgium; catherine.reenaers@chuliege.be; 5 Department of Gastroenterology, AZ Delta, 8800 Roeselare, Belgium; filip.baert@azdelta.be; 6 Department of Gastroenterology, OLV Aalst, 9300 Aalst, Belgium; stijn.vanden.branden@olvz-aalst.be; 7 Department of Gastroenterology, Hopital Erasme, 1070 Brussels, Belgium; anneline.cremer@hubruxelles.be; 8 Department of Gastroenterology, Imelda, 2820 Bonheiden, Belgium; lieven.pouillon@imelda.be; 9 Department of Gastroenterology, AZ Maria Middelares, 9000 Gent, Belgium; pieter.dewint@azdamiaan.be; 10 Department of Gastroenterology, AZ Groeninge, 8500 Kortrijk, Belgium; wouter.vanmoerkercke@azgroeninge.be; 11 Department of Gastroenterology, CHU Namur, 5000 Namur, Belgium; jean-francois.rahier@uclouvain.be; 12 Department of Gastroenterology, UZ Brussel, 1090 Brussel, Belgium; liv.vandermeulen@uzbrussel.be; 13 Department of Gastroenterology, AZ Sint Maarten, 2800 Mechelen, Belgium; jurgen.van.dongen@emmaus.be; 14 Department of Gastroenterology, AZ Sint Lucas, 9000 Gent, Belgium; harald.peeters@azstlucas.be; 15 Department of Gastroenterology, AZ Damiaan, 8400 Oostende, Belgium; guy.lambrecht@azdamiaan.be; 16 Department of Gastroenterology, CHR de la Citadelle, 4000 Liege, Belgium; anne.vijverman@chrcitadelle.be; 17 Department of Pathology, UZ Gent, 9000 Gent, Belgium; amber.vanhaecke@ugent.be (A.V.H.); anne.hoorens@uzgent.be (A.H.)

**Keywords:** ustekinumab, ulcerative colitis, real-life, effectiveness

## Abstract

**Background/Objectives:** Ustekinumab (UST) has shown to be effective and safe in patients with moderate-to-severe UC in the UNIFI trials. However, real-life data on its effectiveness, particularly for histological remission, are still limited. To assess the real-world effectiveness and safety of UST in refractory UC patients. **Methodology:** This multicentric, retrospective cohort study included UC patients treated with UST from September 2020 to June 2023. The primary endpoint was steroid-free clinical remission (partial Mayo score of ≤2 with no subscore > 1) at week 16. Secondary endpoints included clinical, endoscopic, histological response and remission. **Results:** 120 patients with moderate–severe UC were included across 16 centers. Median disease duration was 11 years (1–74 y), and 81 (68%) patients had previously failed ≥2 biological therapies. At week 16, steroid-free clinical remission was achieved in 34% (41/120) of patients, with endoscopic and histological remission in, respectively, 19% (23/120) and 8% (3/37). By week 52, 44% (38/85) of patients were in steroid-free clinical remission, with endoscopic and histological remission, respectively, in 25% (13/52) and 11% (5/45). Active smoking was a negative predictor for steroid-free remission (OR 0.412, *p* = 0.011). UST drug persistence by week 52 was 70.8%. Active smoking (aOR 3.058, *p* = 0.02), prior vedolizumab non-response (OR 2.592, *p* = 0.03) and a high Nancy baseline score (OR 2.46, *p* = 0.04) were associated with early UST failure. No new safety signals were observed. **Conclusions:** In this real-life cohort, UST shows acceptable remission rates and high treatment persistence in refractory UC patients, with a favorable safety profile.

## 1. Introduction

Ulcerative colitis (UC) is a chronic inflammatory bowel disease (IBD) characterized by a relapsing–remitting course of mucosal inflammation, mainly affecting the colon and rectum [1]. Patients present themselves typically with symptoms of rectal bleeding, diarrhea, urgency, and abdominal pain leading to a significant morbidity, impaired quality of life and increased work absenteeism [2,3]. When conventional therapeutic methods such as 5-aminosalicylic acids (5ASAs) and corticosteroids fail, biological therapy is considered. Agents targeting tumor necrosis factor (TNF) (e.g., infliximab or adalimumab) as well as the anti-integrin inhibitor vedolizumab are frequently used as first-line advanced treatment [1]. However, up to 85% of patients in real-life cohorts [4] fail or become resistant to these treatments. In recent years, newer therapeutic options such as Janus kinase (JAK) inhibitors, Ustekinumab (UST), ozanimod, and mirikizumab have emerged, offering alternative mechanisms of action for refractory UC with their own differences in side-effects and efficacy.

UST is a humanized monoclonal antibody that targets the shared p40 subunit of interleukin (IL)-12 and IL-23, pivotal cytokines in the inflammatory pathways of IBD. Initially approved for Crohn’s Disease in 2016, UST received European Medicine Agency approval for moderate-to-severe UC in 2019, with reimbursement in Belgium in 2020 [5]. The pivotal UNIFI trials demonstrated its efficacy and safety for induction and maintenance of remission in 961 UC patients [6]. The long-term extension studies further strengthened the evidence for the applicability of UST in UC [7]. While clinical trials like UNIFI provide high-quality data, they often involve highly controlled settings with stringent inclusion criteria, potentially limiting generalizability to real-world clinical practice. Real-world data are essential for understanding UST’s performance in diverse patient populations. Additionally, histological remission—associated with lower relapse rates and a reduced colorectal cancer risk—has emerged as an aspirational therapeutic target, yet real-world data on this outcome remain sparse [8].

This multicentric, retrospective study aimed to evaluate the real-world short- and long-term effectiveness and safety of UST in UC patients across Belgian centers. In addition, the effects of anti-IL12/23 on histological remission were investigated.

## 2. Methods

### 2.1. Patient Inclusion and Study Design

This multicentric retrospective cohort study included UC patients treated with UST in 16 centers in Belgium from September 2020 to June 2023. Eligible patients were ≥18 years of age at treatment initiation, diagnosed with UC, and had received at least one intravenous dose of UST. Patients with Crohn’s Disease, undifferentiated IBD (IBD-U), or an ostomy were excluded.

### 2.2. Data Collection and Outcomes

Data were extracted from medical records, including demographics, disease characteristics, treatment history, and clinical outcomes. Histological slides were examined by local and/or central pathologists (AH and AVH) in a blinded manner if Nancy index scores were not initially reported. Missing data, especially in secondary outcomes such as biochemical and histological markers, were addressed through sensitivity analyses to minimize bias.

The primary endpoint of the study was steroid-free clinical remission at week 16 defined as a partial Mayo score ≤ 2 with a rectal bleeding subscore of 0, no subscore > 1 and the absence of corticosteroid therapy. Secondary endpoints included clinical, endoscopic, histological and biochemical remission and/or response at different timepoints during the study (week 8, W16, W52 and/or end of treatment). Clinical response was defined as a decrease in partial Mayo score from baseline by ≥3 points and ≥30%, with a decrease in rectal bleeding score ≥ 1 or an absolute rectal bleeding score ≤ 1. Definition of endoscopic remission was an endoscopic Mayo score of 0, while Mayo score ≤ 1 equaled endoscopic response. Biochemical remission was defined as C-reactive protein (CRP) < 5 mg/L or fecal calprotectin < 250 mg/kg, while a decrease in CRP or calprotectin ≥ 50% was defined as a biochemical response. Histological response was defined as a Nancy score of ≤1 and remission as a Nancy score of 0.

Other secondary outcomes included adverse events such as hospitalizations (IBD and non-IBD related), serious infections, mortality, and the occurrence of malignancy during study follow-up.

### 2.3. Statistical Analyses

All analyses were performed in SPSS statistics version 28 (IBM Corp. Released 2020. IBM SPSS Statistics for Mac, Version 27.0, Armonk, NY, USA). GraphPad Prism 10^®^ (GraphPad version 10, GraphPad Software Inc., San Diego, CA, USA) was used for graphical representations of data. Descriptive statistics were presented as means ± standard deviations (SDs) for continuous variables with a normal distribution, medians with interquartile ranges (IQRs) for data with a non-normal distribution and percentages for categorical variables. Chi-square or Fisher’s exact test were performed to compare categorical variables between the different timepoints, and continuous variables were analyzed with the independent sample *t*-test (or Mann–Whitney U test in case of non-normal distribution). Drug persistence over time was illustrated using a Kaplan–Meier curve. Predictors of drug persistence were assessed by Cox regression analysis. Predictors of other endpoints were assessed by uni- and multivariable logistic regression and reported as odds ratios (ORs) and adjusted odds ratios (aORs), respectively, with 95% confidence intervals. A two-tailed *p* < 0.05 was considered statistically significant.

### 2.4. Ethical Statement

The study protocol was reviewed and approved by the Ethical committee of the University Hospital of Ghent (EC2019-0978) as the coordinating center and obtained approval per requirements for each of the participating centers, following the Declaration of Helsinki.

### 2.5. Data Availability Statement

The data that support the findings of this study are available from the corresponding author upon reasonable request.

## 3. Results

### 3.1. Characteristics of the Study Population

This multicentric retrospective cohort study included 120 patients with a median disease duration of 11 years. Demographics and clinical characteristics at baseline are presented in Table 1. The majority started UST for left-sided or extensive colitis, while 6.7% received it for proctitis. Most UC patients (94.2%) had previously failed at least one biological treatment, with 68% failing two or more. Most patients were anti-TNF experienced (80.8%) or had previously been treated with vedolizumab (67.5%), while 35.8% had failed one of the more novel therapies such as JAK-inhibitors (18.3%) or had participated in a clinical trial (17.5%). The majority of patients (88.3%) initiated UST due to moderate-to-severe UC activity, while 10% were treated for pouchitis following prior ileal pouch–anal anastomosis (IPAA). Six patients (5%) initiated UST primarily for associated conditions, such as psoriasis (3.3%) and hidradenitis suppurativa (1.7%).

Comorbidities were present in 34.2% of the cohort, with diabetes (3.3%) and cardiovascular disease (8.3%) being the most common. Eight patients (8.3%) had a history of malignancy, including two patients with active malignancy at the start of UST treatment. Extra-intestinal manifestations were observed in 15.8% of patients, with the most common being psoriasis (10.0%). Seven patients (5.8%) had concomitant primary sclerosing cholangitis (PSC) of whom three patients had undergone a liver transplantation prior to UST treatment start (Table 1).

### 3.2. Effectiveness of Ustekinumab in Ulcerative Colitis

#### 3.2.1. Clinical Remission and Response

**Figure 1:**
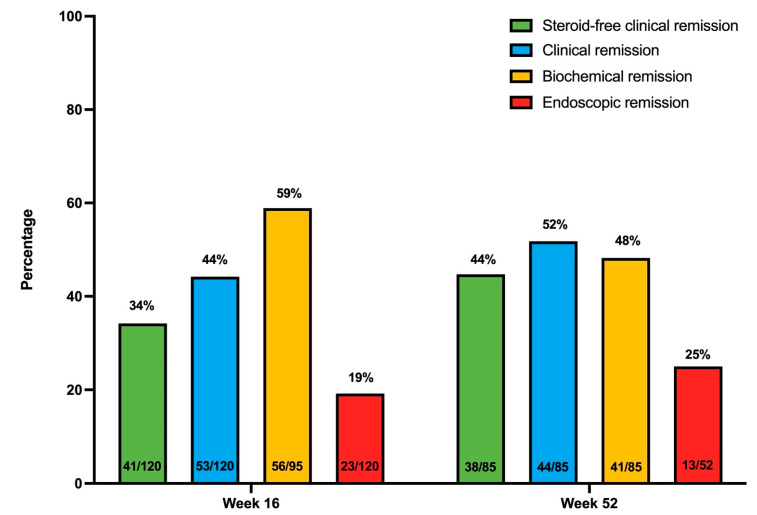
The proportion of ulcerative colitis patients treated with Ustekinumab and in clinical, biochemical, or endoscopic remission at week 16 and week 52.

**Figure 2:**
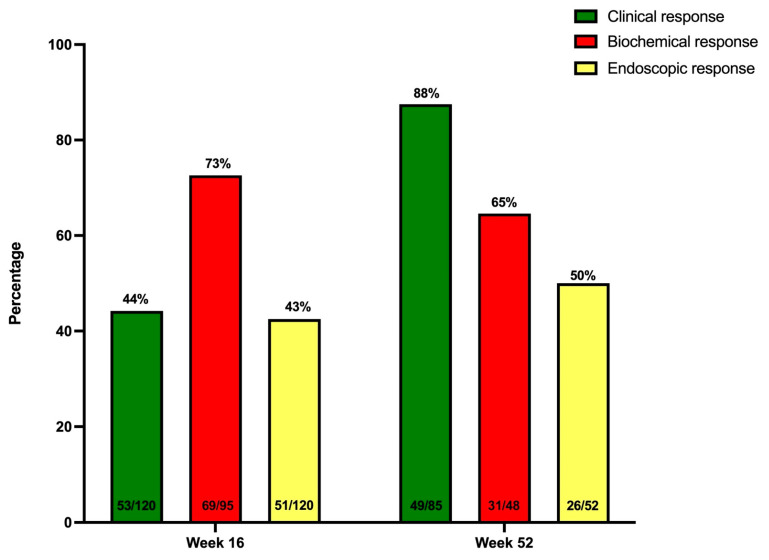
The proportion of UC patients treated with UST and showing a clinical, biochemical, or endoscopic response at week 16 and week 52.

The primary endpoint of steroid-free clinical remission at week 16 was achieved in 34.2% (41/120) of UC patients. By week 52, this rate improved to 44.7% (38/85) among patients still receiving UST. Additionally, 72.5% (87/120) of patients achieved a clinical response by week 16 and 87.5% (49/85) by week 52 (Figure 1 and Figure 2).

The median partial Mayo score decreased significantly from 6 (IQR 5–8) at baseline to 2 (IQR 0–4, *p* < 0.001) at week 16 and 1 (IQR 0–5, *p* < 0.001) at week 52 (Supplementary Figure S1A). In a univariate logistic regression model, only active smoking was found to be a statistically significant negative predictor for achieving steroid-free clinical remission at week 16 (OR 0.412 (0.207–0.819), *p* = 0.011) (Supplementary Table S1).

#### 3.2.2. Biochemical Outcomes

Biochemical remission was observed in 58.9% (56/95) of patients at week 16 and 48.2% (41/85) at week 52, although these outcomes were limited by missing data (20.8% at week 16 and 41.2% at week 52) (Figure 1 and Figure 2). Median CRP levels decreased significantly from 9.8 mg/L (IQR 1.30–13.3) at baseline to 2.4 mg/L (IQR 1.20–6.00; *p* < 0.001)) at week 16 and 3 mg/L (IQR 1.00–5.80); *p* = 0.002) at week 52. Similarly, median fecal calprotectin levels dropped from 965 mg/kg (IQR 144–2354) at baseline to 391 mg/kg (IQR 186–1240; *p* = 0.004) at week 16 and 143 mg/kg (IQR 54–961; *p* < 0.001) at week 52 (Supplementary Figure S2).

#### 3.2.3. Endoscopic Outcomes

Endoscopic remission was achieved in 19.2% (23/120) of patients at week 16 and increased to 25% (13/52) by week 52. Endoscopic response was observed in 42.5% (51/120) at week 16 and 50% (26/52) at week 52 (Figure 1 and Figure 2). The median endoscopic Mayo score improved from 3 (IQR 2–3) at baseline to 1 (IQR 1–3, *p* < 0.001) at week 16 and 1.5 (IQR 0.25–3, *p* < 0.001) at week 52 (Supplementary Figure S1B). In the logistic regression model, prior exposure to vedolizumab was a negative predictor for attaining endoscopic remission at week 16, even after correction for other covariables (aOR 0.139 (0.024–0.787), *p* = 0.026) (Supplementary Table S2).

#### 3.2.4. Histological Outcomes

**Figure 3:**
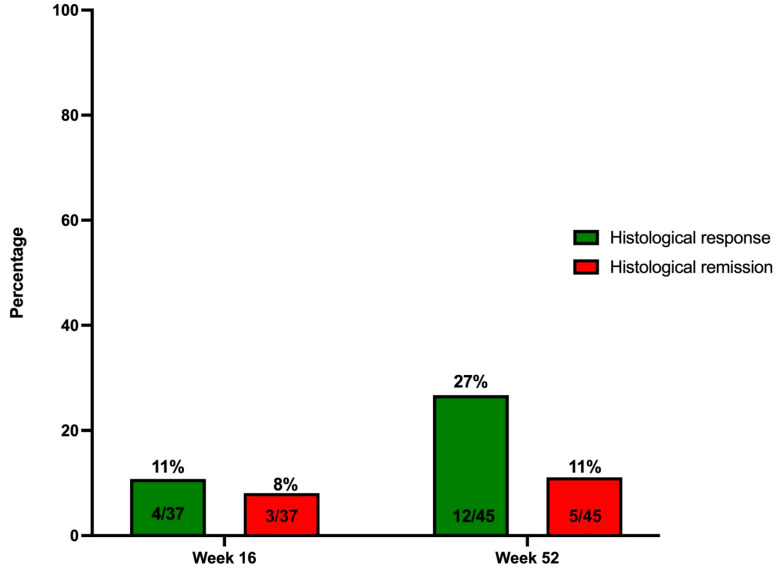
The proportion of ulcerative colitis patients treated with Ustekinumab and showing a histological response (Nancy score ≤ 1) and histological remission (Nancy score = 0) at week 16 and week 52.

Histological remission was achieved in 8.1% (3/37, 69% missing values) of patients at week 16 and 11.1% (5/45, 62% missing) by week 52 (Figure 3). Two patients (40%) in histological remission at W52 also had an endoscopic Mayo 0 score, while three had a Mayo 1 score (60%) at the time of evaluation. Histological response was observed in 10.8% (4/37) and 26.7% (12/45) of patients at week 16 and 52, respectively. No statistically significant predictors could be identified in a logistic regression model (Supplementary Table S3).

### 3.3. Ustekinumab Persistence and Need for Dose Optimization

**Figure 4:**
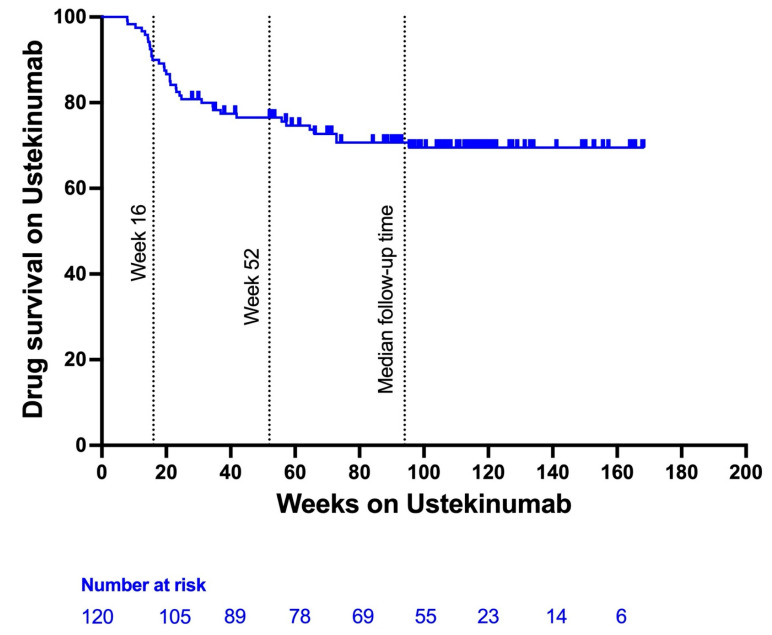
Kaplan–Meier Curve illustrating Ustekinumab persistence in 120 patients with ulcerative colitis. Median follow-up time is 94 weeks (interquartile range 38–116).

UST persistence was 70.8% at one year. Reasons for discontinuation were primary non-response in 15%, while 8% had a secondary loss of response. In two patients (1.7%) UST was discontinued because of adverse events (Figure 4). In a univariate Cox regression analysis, smoking (OR 1.584 (1.032–2.430), *p* = 0.035); prior vedolizumab non-response (OR 2.592 (1.076–6.244), *p* = 0.034) and baseline Nancy score (OR 2.466 (1.068–5.695), *p* = 0.035) were significantly associated with early UST termination. In a multivariate analysis, only smoking status remained statistically significant (aOR 3.058 (1.193–7.836), *p* = 0.020) (Table 2). Sequence of therapy did not significantly affect UST persistence or effectiveness (Supplementary Table S4); however, numerically patients who were prescribed UST in first line (N = 7) continued UST throughout the duration of the study (Supplemental Figure S3).

During the study, 12 patients (10%) required a reinduction dose of UST and/or shortening of the dosing interval. Of these 67% (8/12) recaptured effect and continued UST treatment, while 50% (6/12) achieved steroid-free clinical remission at week 52 and 16.7% (2/12) were in endoscopic remission at the end of follow-up.

### 3.4. Effect on Pouchitis

In this study, twelve patients were treated with UST because of pouchitis (10%), two of them (16.7%) achieved steroid-free clinical remission by week 16, and in one patient, endoscopic remission was seen (8.3%). Five patients (41.7%) experienced a clinical response by week 16. At the end of treatment, five patients (41.7%) continued UST treatment. In these patients, steroid-free clinical and endoscopic remission was achieved in three (25%).

### 3.5. Effect on Extra-Intestinal Manifestations and Concurrent Immune Mediated Inflammatory Diseases

Most common extra-intestinal manifestations at baseline were rheumatological in nature. Nine patients reported pre-existing peripheral arthralgias (7.5%) and eight had active spondyloarthropathy (6.7%, five (4.1%) axial and three peripheral disease (2.5%)). Following 16 weeks of treatment with UST, an improvement of the pre-existing arthralgia was seen in five (55%) patients, while one patient (12.5%) reported an improvement of the axial spondyloarthropathy (Supplementary Figure S4).

Seven patients reported active psoriasis at baseline (5.8%), of which four patients (57%) reported an improvement following 16 weeks of UST treatment. No effect was seen on primary sclerosing cholangitis. No other new onset extra-intestinal manifestations were diagnosed during the follow-up of this study.

### 3.6. Safety of Ustekinumab in Ulcerative Colitis

During the median follow-up period of 94 weeks (38–116), five infectious episodes requiring hospitalization were reported (Table 3). Toxic/allergic adverse events were reported in four patients with elevated liver enzymes being most frequently seen. None of these events required cessation of UST treatment. Eleven patients were hospitalized due to a UC flare. During follow-up, two patients were diagnosed with a new active malignancy (one cholangiocarcinoma and one basal cell carcinoma), neither of which necessitated UST termination.

During follow-up, no new extra-intestinal manifestations were diagnosed in this cohort while being treated with UST, apart from six patients (5%) reporting a new occurrence of arthralgia. In all but one patient, the arthralgia was transient and did not necessitate a change in UST therapy. One pregnancy was documented during this retrospective study and remained uncomplicated despite UST continuation.

## 4. Discussion

In this nation-wide, multicentric real-life effectiveness study in 120 patients with highly refractory ulcerative colitis, we observed high UST drug persistence both after induction (i.e., week 16) and after one year of treatment (respectively, 90 and 71%). The primary endpoint of steroid-free clinical remission at week 16 was achieved in 34%, while 19% of patients were in endoscopic remission, and 8% showed histological remission at this timepoint. These remission rates further improved to, respectively, 45%, 25% and 11% after one year of treatment.

In the pivotal phase III UNIFI trial’s registration trial, the primary endpoint of combined clinical and endoscopic remission after induction (measured at week 8) was seen in 15.5% of the 961 UC patients enrolled. At the end of the trial (week 52), the primary endpoint was achieved in 43.8% of patients [6,9]. The drug persistence rate in the UNIFI study was higher compared to our real-life study (87% vs. 71%). In contrast to our study, however, only patients responding to induction therapy were enrolled in the maintenance phase of the UNIFI trial. Moreover, 49% of patients enrolled in the registration study were bio-naïve whereas only 5.8% of UC patients where not previously exposed to biologicals or JAK inhibitors in this study [6]. Both factors may explain the difference in outcomes and drug persistence rates between the UNIFI trial and our study and further emphasize the discrepancies between clinical trials and the real-world clinical setting.

Effectiveness studies are important in helping us understand how drugs perform in the real world, although only few studies regarding UST in ulcerative colitis have been published. To the best of our knowledge, our study is one of the largest studies, with an extensive follow-up period published to date [10,11]. In a study by the GETAID, Fumery et al. reported steroid-free clinical remission in 32% in a population of 103 highly refractory UC patients with an UST persistence rate of 58% after 52 weeks [12]. A study in tertiary centers from the USA, including 66 UC patients, showed a clinical remission rate of 45% after one year of treatment [13]. In an Italian multicenter study with a similar refractory population of 68 UC patients, 50% of patients achieved steroid-free clinical remission by week 52 with 22% showing mucosal healing and a drug persistence of 87% [14]. A study from the ENEIDA registry in 95 UC patients reported clinical remission rates of 35% and 33% by week 16 and 52, respectively [15]. In another study across four Spanish referral hospitals by Iborra et al., 108 UC patients were included with clinical remission rates of 57% and 69% at week 16 and 52, respectively [16]. In a multicenter cohort consortium from the USA in 245 patients, steroid-free clinical remission was 29% after 6 months [17]. Lastly, a large cohort from the Swedish inflammatory bowel disease quality registry (SWIBREG) reported clinical remission rates of 17% at week 16 with a drug persistence rate of 67% in 133 UC patients with a median follow-up time of 32 weeks [18].

In the study presented here, active smoking had a negative impact on achieving steroid-free clinical remission after induction, while prior failure of vedolizumab was a negative predictor for mucosal healing even after correction for other variables. Few other studies have been able to identify predictors associated with remission [10,11]. In the EINEIDA registry study by Chaparro et al. an elevated CRP (above the normal range) at baseline was associated with a lower probability of achieving remission at week 16 (OR 0.3 (0.1–0.7), *p* < 0.05) [15]. Deepak et al. identified a lower rate of remission in obese patients (BMI ≥ 30) (OR 0.25 (0.08–0.8, *p* = 0.02) [11]. Lastly, in the study by Hong et al., prior anti-TNF primary non-response was shown to be a negative predictor for achieving remission with UST at week 16 (OR 0.03 (0.01–0.82), *p* = 0.04) [13]. Several predictors associated with UST drug persistence at week 52 were identified in this study; active smoking, prior vedolizumab non-response and a high baseline Nancy score were all predictors for UST failure. Active smoking remained a negative predictor even after correction for other covariables indicating the importance of environmental factors in the managing of UC. Although active smoking has been identified as a negative influence on the effectiveness of anti-TNF and vedolizumab, no other studies have investigated the impact of smoking status on UST treatment [19]. Of note, the absolute number of active smokers participating in this study was low, reducing the ability to make a strong recommendation. Prior non-response to vedolizumab and a high baseline Nancy score highlight a higher chance of UST failure in patients with a more severe disease burden. Of note, in Belgium, reimbursement criteria stimulate the first-line use of vedolizumab (e.g., 81% of patients in this study had received vedolizumab prior to inclusion), which might play a role in these findings and highlights the importance of thoughtful sequencing of biologicals. In the Swedish registry study by Thunberg et al., male sex was the only predictor for early UST termination (aOR 4.00 (1.35–11.83), *p* = 0.01) [10,11,18].

During the study, 12 patients (10%) required a reinduction dose of UST and/or shortening of the dosing interval. Of these, 67% (8/12) experienced a recaptured effect and continued UST treatment, while 50% (6/12) achieved steroid-free clinical remission at week 52 and 16.7% (2/12) were in endoscopic remission at the end of follow-up.

Dose optimization, either through IV reinduction and/or interval shortening, was required in 10% of patients, of whom two-thirds had a recaptured response. These decisions were made at the discretion of the treating physician and were not guided by therapeutic drug monitoring (TDM). The pharmacokinetics of UST have been well characterized in both Crohn’s Disease and UC, showing similar profiles across IBD subtypes [20]. UST has a high bioavailability after SC administration, with steady-state concentrations typically achieved around 8 weeks after the first SC dose. Although TDM is not yet routinely implemented in clinical practice for UST, several studies have demonstrated an association between serum UST concentrations and clinical outcomes. Notably, shortening the dosing interval from every 8 weeks to every 4 weeks results in an approximate four-fold increase in serum trough levels, supporting the rationale for dose intensification in selected patients [20,21].

Real-life data regarding induction of histological remission in UC patients treated with UST is sparse [10,11]. In the UNIFI trial, histological improvement (defined as neutrophil infiltration in <5% of crypts, no crypt destruction, and no erosions, ulcerations, or granulation tissue) was seen in 36.8% and 56.7% of UC patients after induction and week 52, respectively [6]. None of the other real-life effectiveness studies report histological data [12,14,15,16,17,18]. Hong et al. report histological remission in four of twelve patients with available data by week 52 [13]. Additionally, in a smaller Belgian cohort of 42 UC patients, histological remission (defined as Nancy score = 0) was seen in 45% of patients by week 16 [22]. In this study, we showed that histological remission in UC patients after one year of UST treatment is achieved in 11% of patients, while a histological response (defined as a Nancy score ≤ 1) was seen in 26%. More real-life data on histological remission/response rates are necessary as both were stated as aspirational goals associated with lower relapse rates and an overall lower risk of developing colorectal cancer [8].

A subgroup of the UC patients included in this study previously underwent an ileal pouch–anal anastomosis (IPAA) and were treated with UST because of a flare of steroid-resistant pouchitis (N = 12) with steroid-free clinical remission induced in 17% and endoscopic remission in 8% by week 16. UST persistence in this group of patients was 42% after one year of treatment. Despite not being within the scope of this study, it adds to the sparse data available for treating steroid-refractory pouchitis with UST. One other study by Outtier et al. showed steroid-free remission rates of 27% in 22 pouchitis patients [23].

During the median follow-up time of 94 weeks (38–116), no important new safety signals associated with UST treatment were observed. Described side effects of UST in the UNIFI studies include nasopharyngitis and upper respiratory tract infections. In patients with pre-existing rheumatological extra-intestinal manifestations, no exacerbation of symptoms was seen, although 5% of patients reported transient arthralgias that disappeared and did not necessitate an alteration in therapy. Other studies specifically investigating the occurrence of extra-intestinal manifestations in IBD patients treated with UST showed similar findings [24,25]. On other IMIDs, UST had a positive effect particularly on psoriasis.

Because of its retrospective design, our study has several limitations. Firstly, the lack of a control group presents a major limitation and precludes any firm conclusions about the effects of UST in treating UC patients. Secondly, the variability in follow-up time might hamper the generalizability of some findings contrasting with the standardized follow-up in a clinical trial setting. Lastly, due to the retrospective observational design outcome assessments were neither standardized nor mandatory and varied considerably between patients. This is especially true for biochemical data (e.g., fecal calprotectin measurements are not reimbursed for UC patients in Belgium) and endoscopy, but also for the histological data (lack of central reading) and thus might affect the generalizability of these findings. Furthermore, these missing data might have introduced bias as patients with more severe activity might have a more stringent follow-up and thus more data available. Despite the shortcomings, this study also has several strengths. Being a multicentric study with 16 participating centers, both secondary and tertiary across Belgium, it accounts for any therapeutic variability across the country and represents the real-life situation. Secondly, the long follow-up time (median of 94 months) provides a good insight in the treatment of a life-long disease.

In conclusion, this multicentric real-life effectiveness study in patients with refractory ulcerative colitis shows acceptable remission rates, a good safety profile and a relatively high drug persistence rate of UST, validating its role as a possible treatment strategy in ulcerative colitis patients.

## Figures and Tables

**Table 1:** Baseline characteristics of the Ustekinumab-treated patients with ulcerative colitis (n = 120).

Age (years, range)	44.5 (19–89)
**Sex** (n, %)	
Female	61 (50.8%)
Male	59 (49.2%)
**Disease duration** (years, range)	11 (1–74)
**Smoking** (n, %)	
Current smoker	5 (4.2%)
Never smoked	81 (67.5%)
Former smoker	34 (38.4%)
**Disease extent** (n, %)	
Proctitis (Montreal E1)	8 (6.7%)
Left-sided colitis (Montreal E2)	69 (57.5%)
Extensive colitis (Montreal E3)	43 (35.8%)
**Comorbidities** (n,%)	41 (34.2%)
Multiple sclerosis	1 (0.8%)
Diabetes mellitus	4 (3.3%)
Cardiovascular comorbidity	10 (8.3%)
Malignancy	10 (8.3%)
Colon cancer	2 (1.6%)
Non-melanoma skin cancer	3 (2.5%)
Other solid organ cancer	5 (4.1%)
**Active malignancy at start UST**	2 (1.6%)
**Extra-intestinal manifestations** (n,%)	19 (15.8%)
PSC	7 (5.8%)
Spondylarthropathy - Axial - Periferal	8 (6.6%) 5 (4.1%) 3 (2.5%)
Uveitis	2 (1.6%)
Erythema nodosum	2 (1.6%)
**Other concurrent IMIDs**	
Psoriasis	12 (10.0%)
Hydradinitis suppurativa	1 (0.8%)
**BMI** (kg/m^2^) (median, range)	24 (14–38)
**IBD hospitalization in previous 12 months** (n, %)	27 (22.5%)
**IPAA** (n, %)	12 (10.0%)
**Previous anti-TNF exposure** (n, %)	97 (80.8%)
Infliximab	77 (65.0%)
Adalimumab	49 (40.8%)
Golimumab	14 (11.9%)
**Previous vedolizumab use** (n, %)	81 (67.5%)
**Previous JAK-inhibitor use** (n, %)	22 (18.3%)
**Previous cyclosporine use** (n, %)	6 (5.0%)
**Previous FMT** (n, %)	3 (2.5%)
**Previous clinical trial**	18 (15.0%)
**Failed biologicals**	
Biological naive	7 (5.8%)
1 biological	32 (26.7%)
2 biologicals	43 (35.8%)
≥3 biologicals	38 (31.6%)
**Failed anti-TNF**	
Anti-TNF naive	23 (19.2%)
1 anti-TNF failed	59 (49.2%)
2 anti-TNFs failed	33 (27.5%)
3 anti-TNFs failed	5 (4.2%)
**Concurrent immunomodulator use**	14 (11.9%)
Azathioprine	10 (8.3%)
Methotrexate	4 (3.3%)
**Concurrent use of corticosteroids at baseline**	65 (54.2%)

**Table 2:** Baseline predictors of early Ustekinumab cessation in patients with ulcerative colitis (n = 120).

	Univariable Analysis		Multivariable Analysis	
	OR (95% CI) *p*-Value		OR (95% CI) *p*-Value	
Sex (male)	0.899 (0.463–1.745)	0.753	1.380 (0.422–4.517)	0.594
Age	0.991 (0.969–1.014)	0.447	0.992 (0.958–1.026)	0.624
Disease extent				
Proctitis or left-sided colitis	Reference		Reference	
Pancolitis (E3)	1.435 (0.728–2.877)	0.297	1.089 (0.379–3.132)	0.874
Smoking	1.584 (1.032–2.430)	**0.035**	3.058 (1.193–7.836)	**0.020**
Prior medical therapy				
Anti-TNF	0.786 (0.357–1.730)	0.549	2.594 (0.299–22.486)	0.387
Vedolizumab	2.592 (1.076–6.244)	**0.034**	1.761 (0.525–5.906)	0.360
JAK-I	1.100 (0.480–2.519)	0.821	0.885 (0.291–2.691)	0.829
Disease severity at baseline	1.274 (0.749–2.167)	0.372	2.010 (0.575–7.030)	0.274
Nancy score at baseline	2.466 (1.068–5.695)	**0.035**	2.305 (0.846–6.279)	0.102

Note: bold values denote statistical significance at *p* < 0.05 level by a logistic regression model. Abbreviations: CI—confidence interval; OR—odds ratio.

**Table 3:** Overview of adverse effects during Ustekinumab treatment in ulcerative colitis patients.

Adverse Events (n, %)	
Infectious	5 (4.2%)
Clostridium difficile infection	1 (0.8%)
Herpes zoster	1 (0.8%)
Peri-anal abscess	1 (0.8%)
Candidiasis	1 (0.8%)
COVID-19 infection	1 (0.8%)
UC-related hospitalization	11 (9.2%)
Malignancy	2 (1.6%)
Basal cell carcinoma	1 (0.8%)
Cholangiocarcinoma	1 (0.8%)
Athralgia	6 (5.0%)
Toxic/allergic	4 (3.3%)
Elevated liver enzymes	2 (1.6%)
Urticaria	1 (0.8%)
Local reaction to injection	1 (0.8%)

## Data Availability

The data presented in this study are available on request from the corresponding author.

## Supplementary Materials

The following supporting information can be downloaded at: https://www.mdpi.com/article/10.3390/jcm14186506/s1, Figure S1: Evolution of the partial Mayo score (A) and endoscopic Mayo score (B) in ulcerative colitis patients treated with Ustekinumab; Figure S2: Evolution of C-Reactive Protein (CRP, mg/L) and calprotectin (mg/kg) in ulcerative colitis patients treated with Ustekinumab; Figure S3: Kaplan-Meier Curve illustrating Ustekinumab persistence in 120 patients with ulcerative colitis according to Ustekinumab treatment line. Median follow-up time UST first line group 116 weeks (85.587-147.413), second line UST 97.798 (80.407-115.118), third line UST (124.282 (110.004-138.559); Figure S4: Patients with active symptoms of extra-intestinal manifestations in ulcerative colitis patients treated with Ustekinumab; Table S1: Baseline predictors of steroid-free clinical remission at week 16 in ulcerative colitis patients treated with Ustekinumab; Table S2: Baseline predictors of endoscopic remission at week 16 in ulcerative colitis patients treated with Ustekinumab; Table S3: Baseline predictors of histological remission at week 52 in ulcerative colitis patients treated with Ustekinumab; Table S4: Effect of first line therapy on Ustekinumab effectiveness at week 16 in ulcerative colitis patients.

## Abbreviations

The following abbreviations are used in this manuscript:

5ASA	5-Aminosalicylic Acid
aOR	Adjusted Odds Ratio
CRP	C-reactive protein
IBD	Inflammatory Bowel Disease
IL	Interleukin
OR	Odds Ratio
TNF	Tumor Necrosis Factor
UC	Ulcerative Colitis
UST	Ustekinumab
