# Supplementary material for: Ustekinumab in Ulcerative Colitis: A Real-Life Effectiveness Study Across Multiple Belgian Centers (SULTAN)
Source: J Clin Med. 2025 Sep 16;14(18):6506. doi: 10.3390/jcm14186506 (PMC12471188; doi:10.3390/jcm14186506)
Supplement: Supplementary file 1 [file jcm-14-06506-s001.zip › jcm-3773350-supplementary.pdf]

Journal of Clinical Medicine

MDPI

Supplementary tables

Supplementary Table S1. – Baseline predictors of steroid-free clinical remission at week 16 in ulcerative colitis patients treated with Ustekinumab.

	Univariable analysis		Multivariable analysis	
	OR (95% CI)	*p-value*	*OR (95% CI)*	*p-value*
Sex (male)	1.379 (0.646-2.943)	0.407	0.604(0.144-2.532)	0.604
Age	1.019 (0.995-1.044)	0.113	1.009 (0.969-1.052)	0.655
Disease extent				
Proctitis or left-sided colitis	Reference		Reference	
Pancolitis (E3)	1.628 (0.746-3.550)	0.221	1.089 (0.379-3.132)	0.874
Smoking	0.412 (0.207-0.819)	**0.011**	0.276 (0.076-1.010)	0.052
Prior medical therapy				
Anti-TNF	0.611 (0.241-1.545)	0.298	0.528 (0.099-1.052)	0.655
Vedolizumab	0.642 (0.290-1.419)	0.273	0.481 (0.123-1.878)	0.292
JAK-I	0.675 (0.242-1.882)	0.452	1.665 (0.379-7.316)	0.500
Disease severity at baseline	0.749 (0.434-1.294)	0.301	1.334(0.331-5.372)	0.685
Nancy Score at baseline	0.629 (0.280-1.414)	0.262	0.578 (0.216-1.551)	0.277

Note: bold values denote statistical significance at p<0.05 level by logistic regression model

Abbreviations: CI, confidence interval; OR, odds ratio.

Supplementary Table S2. – Baseline predictors of endoscopic remission at week 16 in ulcerative colitis patients treated with Ustekinumab.

	Univariable analysis		Multivariable analysis	
	OR (95% CI)	*p-value*	*OR (95% CI)*	*p-value*
Sex (male)	0.862 (0.347-2.140)	0.748	0.864 (0.108-6.981)	0.890
Age	1.004 (0.976-1.032)	0.807	0.980 (0.928-1.035)	0.474
Disease extent				
Proctitis or left-sided colitis	Reference		Reference	
Pancolitis (E3)	2.252 (0.894-5.673)	0.085	1.299 (0.185-9.108)	0.792
Smoking	0.546 (0.250-1.192)	0.129	0.685 (0.207-2.261)	0.534
Prior medical therapy				
Anti-TNF	1.157 (0.352-3.800)	0.810	0.417 (0.051-9.108)	0.416
Vedolizumab	0.354 (0.139-0.897)	**0.029**	0.139 (0.024-0.787)	**0.026**
JAK-I	0.357 (0.079-1.696)	0.199	0.477 (0.037-6.063)	0.568
Disease severity at baseline	0.667 (0.362-1.230)	0.195	1.517(0.202-11.411)	0.686
Nancy Score at baseline	0.548 (0.219-1.370)	0.198	0.402 (0.120-1.344)	0.139

Note: bold values denote statistical significance at p<0.05 level by logistic regression model

Abbreviations: CI, confidence interval; OR, odds ratio.

Supplementary Table S3. – Baseline predictors of histological remission at week 52 in ulcerative colitis patients treated with Ustekinumab.

	Univariable analysis		Multivariable analysis	
	OR (95% CI)	*p-value*	*OR (95% CI)*	*p-value*
Sex (male)	0.862 (0.276-12.191)	0.531	0.208 (0.007-6.398)	0.369
Age	1.053 (0.995-1.115)	0.074	1.062 (0.973-1.160)	0.175
Disease extent				
Proctitis or left-sided colitis	Reference		Reference	
Pancolitis (E3)	1.111 (0.166-7.431)	0.913	0.491 (0.028-8.712)	0.628
Smoking	0.428 (0.055-3.303)	0.416	0.625 (0.007-12.368)	0.998
Prior medical therapy				
Anti-TNF	0.848 (0.082-8.792)	0.890	0.653 (0.015-29.224)	0.653
Vedolizumab	0.193 (0.084-9.267)	0.998	0.944 (0.004-9.883)	0.997
JAK-I	0.861 (0.085-8.706)	0.899	1.017 (0.030-34.794)	0.568
Disease severity at baseline	0.750 (0.111-5.060)	0.768	1.167(0.049-27.768)	0.924
Nancy Score at baseline	0.333 (0.092-1.199)	0.092	0.270 (0.030-2.445)	0.244

Note: bold values denote statistical significance at $p<0.05$ level by logistic regression model

Abbreviations: CI, confidence interval; OR, odds ratio.

Supplementary Table S4. – Effect of first line therapy on Ustekinumab effectiveness at week 16 in ulcerative colitis patients.

	No prior biologicals	First line anti-TNF	First line vedolizumab	p-value
N, % total	7 (5.8%)	27 (22.5%)	13 (10.8%)	
Steroid-free clinical remission	4 (57.1%)	11 (40.7%)	6 (46.2%)	0.733
Clinical remission	5 (71.4%)	14 (51.9%)	6 (46.2%)	0.545
Clinical response	6 (85.7%)	21 (77.8%)	7 (53.8%)	0.197
Biochemical remission	2 (28.6%)	14 (51.8%)	6 (46.2%)	0.577
Biochemical response	3 (42,9%)	16 (80%)	6 (46.2%)	0.604
Endoscopic remission	2 (28.6%)	9 (33.3%)	2 (15.4%)	0.493
Endoscopic response	3 (42.9%)	13 (48.1%)	4 (30.8%	0.581
Histological remission	0 (100%)	0 (100%)	0 (100%)	NS
Histological response	0 (100%)	0 (100%)	0 (100%)	NS

Note: bold values denote statistical significance at $p<0.05$ level. Chi-square test.

Supplementary figures

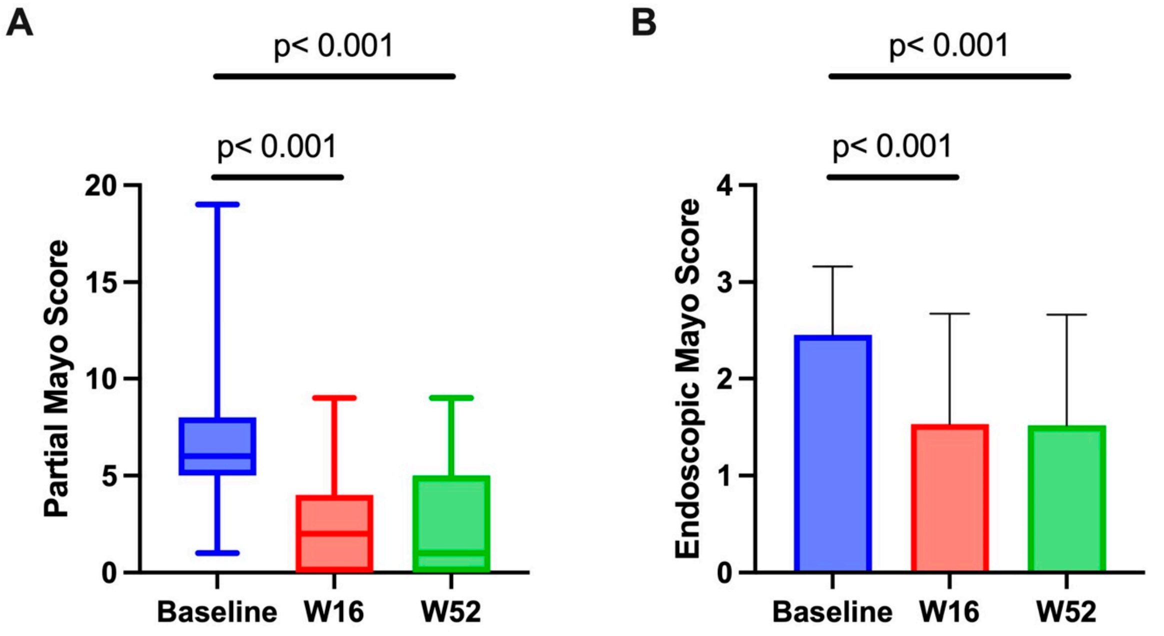

Supplementary Figure S1. – Evolution of the partial Mayo score (A) and endoscopic Mayo score (B) in ulcerative colitis patients treated with Ustekinumab.

***Wilcoxon signed rank test.**

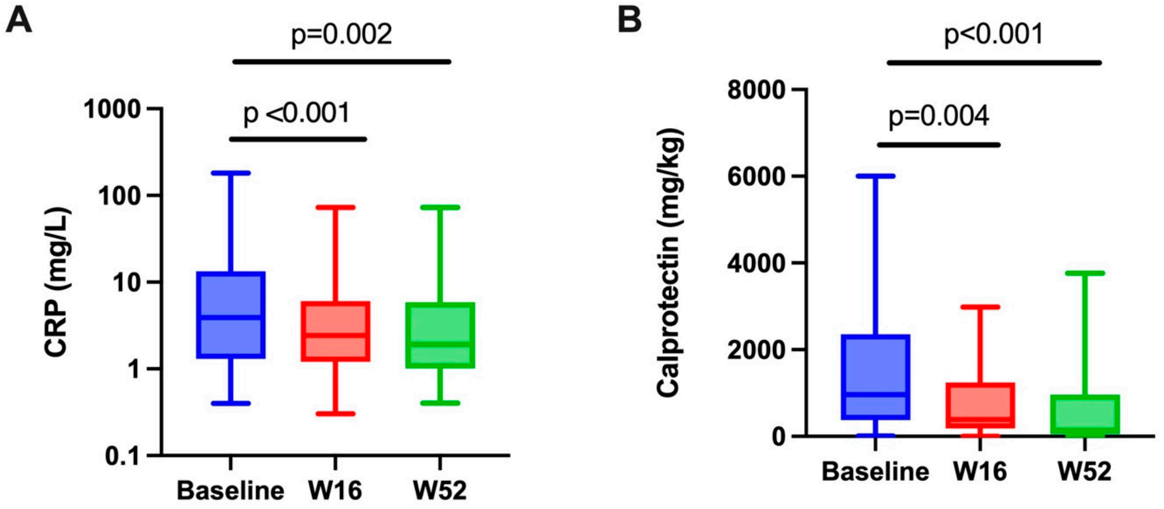

Supplementary Figure S2. – Evolution of C-Reactive Protein (CRP, mg/L) and calprotectin (mg/kg) in ulcerative colitis patients treated with Ustekinumab.

***Wilcoxon signed rank test.**

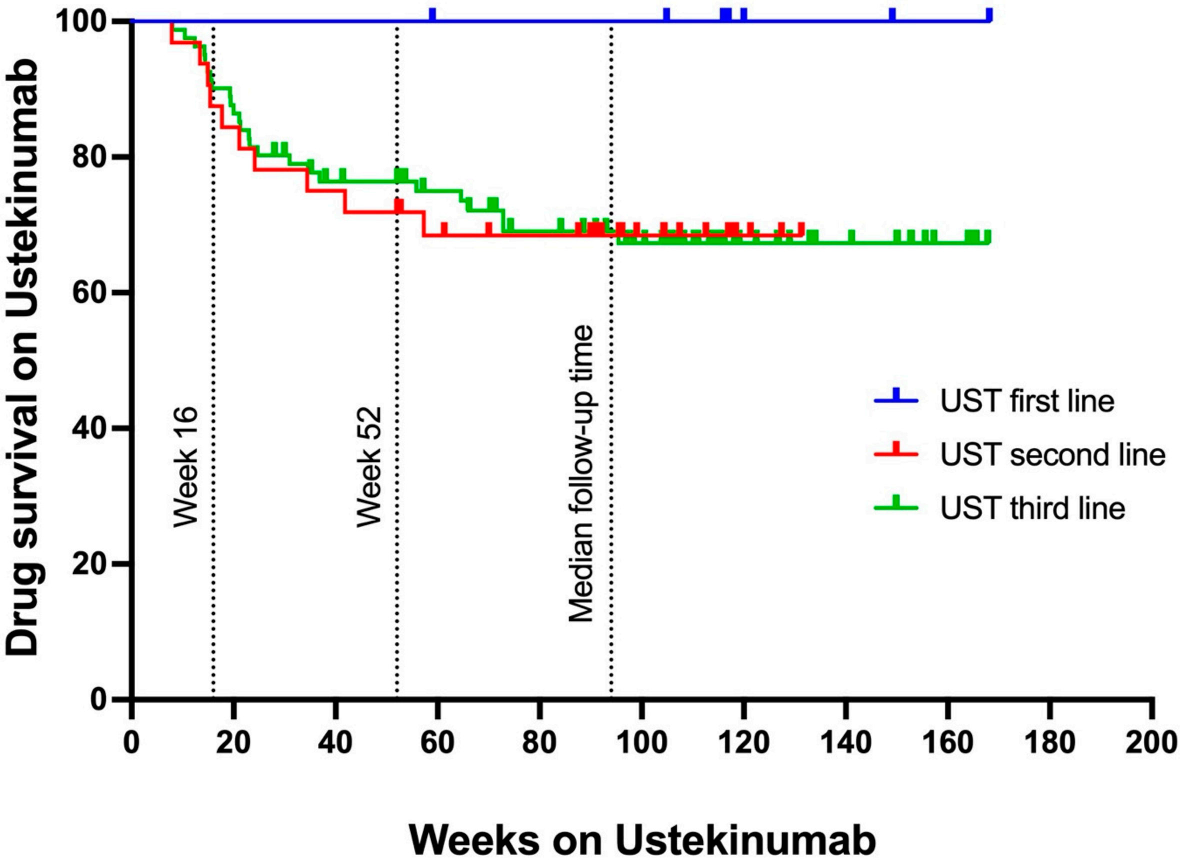

Supplementary Figure S3. – Kaplan-Meier Curve illustrating Ustekinumab persistence in 120 patients with ulcerative colitis according to Ustekinumab treatment line. Median follow-up time UST first line group 116 weeks (85.587-147.413), second line UST 97.798 (80.407-115.118), third line UST (124.282 (110.004-138.559)

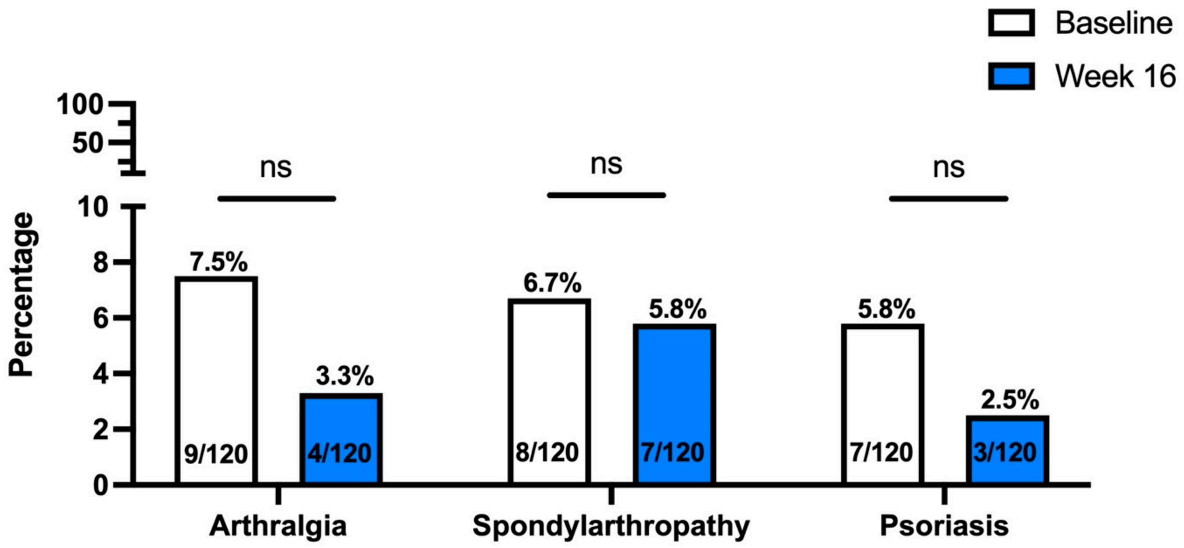

Supplementary Figure S4. – Patients with active symptoms of extra-intestinal manifestations in ulcerative colitis patients treated with Ustekinumab.
